# Analysis and validation of biomarkers and immune cell infiltration profiles in unstable coronary atherosclerotic plaques using bioinformatics and machine learning

**DOI:** 10.3389/fcvm.2025.1451255

**Published:** 2025-02-14

**Authors:** Pengyue Jin, Shangyu Zhang, Li Yang, Yujie Zeng, Yongguo Li, Renkuan Tang

**Affiliations:** ^1^ Department of Forensic Medicine, Faculty of Basic Medical Science, Chongqing Medical University, Chongqing, China; ^2^ Chongqing Engineering Research Center for Criminal Investigation Technology, Chongqing, China; ^3^ Chongqing Key Laboratory of Forensic Medicine, Chongqing, China; ^4^ Department of Anatomy, Faculty of Basic Medical Sciences, Sichuan College of Traditional Chinese Medicine, Mianyang, China

**Keywords:** coronary atherosclerosis, unstable plaque, weighted gene co-expression network analysis, machine learning, biomarkers, immune cell infiltration

## Abstract

**Introduction:**

Decreased stability of coronary atherosclerotic plaques correlates with a heightened risk of acute coronary syndrome (ACS). Thus, early diagnosis and treatment of unstable plaques are imperative in averting adverse cardiovascular events. This study aims to identify diagnostic biomarkers for unstable coronary atherosclerotic plaques and investigate the role of immune cell infiltration in their formation.

**Methods:**

The datasets GSE163154 and GSE111782, obtained from the gene expression omnibus (GEO) database, were amalgamated for bioinformatics analysis, using the dataset GSE43292 as a test set. Sequentially, we performed principal component analysis (PCA), differential gene expression analysis, enrichment analysis, weighted gene co-expression network analysis (WGCNA), utilized a machine learning algorithm to screen key genes, conducted receiver operating characteristic (ROC) curve analysis and nomogram model to assess biomarker diagnostic efficacy, validated the biomarkers, and analyzed immune cell infiltration.

**Results:**

In conclusion, enrichment analyses demonstrate that genes are significantly enriched in inflammatory and immune-related pathways. We identified HSPA2 and GEM as key genes and validated them experimentally. Significant differences existed in immune cell infiltration between subgroups. Additionally, HSPA2 and GEM showed significant associations with a wide range of immune cells.

**Discussion:**

HSPA2 and GEM can function as diagnostic biomarkers for unstable coronary atherosclerotic plaques. In combination with immune cell infiltration analyses, our study provides new insights into the future study of unstable plaque occurrence and molecular mechanisms.

## 1 Introduction

According to the Global Disease and Burden Study, cardiovascular disease remains the leading cause of death worldwide, with approximately 50% of fatalities attributed to ischemic heart disease (1, 2). Each year, approximately 7 million individuals worldwide are diagnosed with acute coronary syndrome (ACS), and over one-third of fatalities in developed nations are linked to this condition (3). Luminal thrombosis resulting from coronary atherosclerotic plaque rupture or plaque surface erosion constitutes the primary cause of ACS episodes. Furthermore, nearly 70% of fatal acute myocardial infarctions and sudden coronary deaths stem from coronary plaque rupture (4). Coronary atherosclerosis, a chronic inflammatory disease, often remains asymptomatic for decades. However, due to plaque lipid deposition, inflammatory cell infiltration, expression of inflammatory cytokines, and impaired clearance of apoptotic cells, it ultimately leads to decreased plaque stability and precipitates adverse cardiovascular events (5). Typical pathological features of unstable plaques include a thin fibrous cap (less than 60 µm), a large necrotic lipid core, inflammatory cell infiltration (particularly at the shoulder of the plaque fibrous cap and at the junction with the surrounding endothelium), and spotty calcification (6–8). Early identification of unstable plaques and prevention of coronary plaque rupture or adverse cardiovascular events are clinically significant because unstable plaques are more prone to causing luminal thrombosis and elevating the risk of ACS.

Previous clinical and experimental evidence suggests that innate and adaptive immune responses play an important role in atherosclerotic plaque progression and plaque instability. Atherosclerosis arises from endothelial cell dysfunction in the lumen at sites of laminar flow shear stress disturbance. As low-density lipoprotein (LDL) enters the subendothelium, where it undergoes oxidation or other chemical modifications, it activates vascular endothelial cells and smooth muscle cells. The release of large amounts of chemokines and adhesion molecules triggers a cascading response of immune cell aggregation (9). Monocyte-derived macrophages initiate subendothelial lipoprotein uptake and transformation into foam cells (10). Uptake of oxidized LDL activates the expression of cytokines such as the NACHT, LRR, and PYD domains-containing protein 3 (NLRP3) inflammasome and Interleukin (IL)-1β in macrophages, further promoting the recruitment of inflammatory cells and inflammatory response in plaques (11). Excessive lipid uptake leads to massive foam cell apoptosis accompanied by impaired macrophage clearance of dead cells resulting in the formation of lipid necrotic cores and the release of large amounts of pro-inflammatory cytokines and matrix metalloproteinases (MMPs), further increasing plaque instability (10, 12, 13). Adaptive immunity plays a pivotal role in either promoting or mitigating atherosclerosis progression. Previous studies have demonstrated that antigen-presenting cells (APCs), predominantly macrophages and dendritic cells, present antigens such as LDL containing apolipoprotein B (ApoB) to adaptive immune cells. This process stimulates their differentiation into various subtypes and elicits pro- or anti-inflammatory effects (14). T helper 1 (Th1) cells secrete pro-inflammatory cytokines, such as interferon-γ (IFN-γ) and tumor necrosis factor-α (TNF-α), which exacerbate the inflammatory response within plaques and promote the expression of MMPs, thereby reducing plaque stability. Conversely, T Regulatory (Treg) cells inhibit plaque inflammation and immune cell activation by expressing anti-inflammatory cytokines such as IL-10 and transforming growth factor-β (TGF-β). Additionally, they promote mesenchymal collagen synthesis to maintain plaque stability (15). Different subtypes of B cells express various antibodies against atherogenic antigens, thereby influencing either anti-atherogenic or pro-atherogenic mechanisms (14).

Weighted gene co-expression network analysis (WGCNA) is a systems bioinformatics algorithm utilized for analyzing large, high-dimensional datasets. It enables the integration of genes with highly correlated expression patterns into gene modules, wherein genes sharing the same module exhibit similar biological functions and regulatory roles. Correlations between modules and disease phenotypes are computed to identify potential biomarkers or therapeutic targets (16). WGCNA has proven successful in identifying key genes associated with atherosclerotic plaque rupture (17). Machine learning is frequently employed in biological research to handle large, complex datasets, constructing predictive models based on underlying algorithms and provided datasets (18). In contrast to prior research (19), our study integrated multiple high-throughput sequencing datasets pertaining to plaque stability for comprehensive analysis. We employed WGCNA in conjunction with various machine learning algorithms to enhance the efficiency and accuracy of biomarker identification, relying on the analysis of differentially expressed genes (DEGs). Additionally, we validated these findings in a test cohort and human tissue samples. Additionally, we delineate the immune cell infiltration associated with plaque stability and investigate the correlation between biomarkers and immune cell infiltration. These findings may contribute to elucidating the pathogenesis of unstable plaques and identifying potential diagnostic biomarkers and therapeutic targets.

## 2 Materials and methods

### 2.1 Data download and pre-processing

Data sets were obtained from the Gene Expression Omnibus (GEO) database (https://www.ncbi.nlm.nih.gov/geo/) by applying filters for “plaque stability,” “atherosclerosis,” “Homo sapiens,” and “microarray-based expression profiling.” Three datasets, namely GSE111782, GSE163154, and GSE43292, were acquired: GSE111782 comprised 9 unstable plaque samples and 9 stable plaque samples, GSE163154 comprised 27 unstable plaque samples and 16 stable plaque samples, and GSE43292 comprised 32 unstable plaque samples and 32 stable plaque samples. The datasets GSE111782, GSE163154, and GSE43292 were retrieved using the R package GEOquery. Gene name conversions were performed based on the platform annotation information corresponding to each dataset. We merged datasets GSE111782 and GSE163154 to form the training set. Batch effects between different datasets were mitigated using the “combat” function in the R package sva to minimize their impact on the analysis results. Subsequently, GSE43292 was designated as the test set.

### 2.2 Pathological sample collection and plaque stability assessment

Coronary artery tissue samples collected from autopsy cases at the Department of Forensic Medicine, College of Basic Medical Sciences, Chongqing Medical University (Chongqing Forensic Injury Institute) between 2012 and 2022 were chosen following the guidelines outlined in the Declaration of Helsinki and the regulations on autopsy provided by the Ministry of Health of the People's Republic of China. Informed consent was acquired from either the donor or the next of kin of the deceased and was sanctioned by the Ethics Review Committee of Chongqing Medical University (approval number: 2024024). Sampling was conducted within 48 h of death in all autopsy cases. Coronary artery tissue samples were preserved in 4% paraformaldehyde solution for 72 h. Vessel cross-sections were embedded in paraffin, and 5-µm sections were subsequently prepared and stained with hematoxylin-eosin. Both naked eye examination and histological analysis revealed the presence of atherosclerosis in the coronary arteries. The histological stability of coronary atherosclerotic plaques was evaluated using the American Heart Association (AHA) atherosclerotic plaque stability scoring system (20–22). This scoring system furnishes a comprehensive score derived from parameters including plaque hemorrhage, thrombosis, lipid core size, fibrous tissue percentage, inflammatory cell infiltration, foam cell count, and plaque rupture to evaluate plaque stability.

### 2.3 Identification and enrichment of DEGs

DEGs in the unstable plaque group compared to the stable plaque group were analyzed in the merged dataset using the R package limma. The thresholds for DEGs were set at |log2 fold change (FC) >1 and *p*-value < 0.05. Volcano maps and heat maps were plotted using the R package ggplot2 and R package heatmap. Kyoto Encyclopedia of Genes and Genomes (KEGG) and Gene Ontology (GO) enrichment analyses of DEGs were performed using the R package clusterProfiler. The GO enrichment analyses consisted of three parts: Biological process (BP), cellular component (CC), and molecular function (MF). Multiple test correction for *p*-values was performed using Benjamini-Hochberg. *p*-value < 0.05 and *q*-value < 0.05 were considered as significant enrichment.

### 2.4 Construction of weighted gene co-expression network

The median absolute deviation (MAD) of genes was initially computed from gene expression profiles, and a weighted gene co-expression network was subsequently established for genes within the top 50% of MAD employing the R package WGCNA. Expression profiles were examined for missing values, and sample clustering trees were constructed to identify significant outlier samples. Subsequently, the “pickSoftThreshold” function was utilized to determine the optimal soft threshold value, ensuring coherence between gene connections in the co-expression network and the scale-free network distribution. A neighbor-joining matrix was created using the optimal soft threshold β, followed by transformation into a topological overlap matrix (TOM). Hierarchical clustering was conducted to group genes with analogous expression patterns into cohesive modules, with a minimum module size established at 30. Module eigengene was computed for each module, and modules exhibiting similarity in the clustering tree were merged according to inter-module correlation. Modules were correlated with phenotypic data, and gene-to-module module membership (MM) and gene significance (GS) between genes and phenotypes within the modules were determined. Module key genes were identified using thresholds of GS >0.2 and MM >0.8, which were subsequently employed for further analyses.

### 2.5 Screening and validation of potential diagnostic biomarkers

By intersecting DEGs with the key module genes derived from WGCNA, 310 genes were identified. Subsequently, two machine learning algorithms were employed to identify characteristic genes. The least absolute shrinkage and selection operator (LASSO) regression model was constructed utilizing the R package glmnet, with the parameter “alpha = 1” configured to introduce a penalty term. As the regularization parameter lambda increases, the regression coefficients of the model variables gradually approach 0. Ten-fold cross-validation was employed to select lambda.min as the optimal value of lambda, yielding ten feature genes corresponding to lambda.min. Random forest (RF) analysis was conducted utilizing the R package randomForest, with the parameter “ntree  = 1,000” determined based on the number of decision trees corresponding to the minimum model error. The top 20 feature genes were selected based on the Mean Decrease Accuracy (MDA) of the model, a method used for gene importance ranking. Heat shock-related 70 kDa protein 2 (HSPA2) and GTP binding protein overexpressed in skeletal muscle (GEM) were identified by intersecting the results of the two machine learning algorithms described above. The Wilcoxon rank sum test was employed to evaluate the statistical significance of differences in biomarker expression between unstable and stable plaques in both training and test sets. The R package pROC was utilized to generate a receiver operating characteristic (ROC) curve, and the area under the curve (AUC) was computed to evaluate the diagnostic efficacy of the biomarkers for unstable plaques in both the training and test cohorts. Using the “rms” and “rmda” package, we constructed a nomogram of the marker genes GEM and HSPA2 based on multifactorial regression analysis, and plotted clinical decision curve analysis (DCA) and calibration curve.

### 2.6 Immune cell infiltration analysis and its correlation with diagnostic biomarkers

Utilizing the R package IOBR (23) for gene expression profiling, we employed the cell-type identification by estimating relative subsets of RNA transcripts (CIBERSORT) algorithm to evaluate the 22 levels of immune cell infiltration within atherosclerotic plaques. We computed Spearman correlations between immune cells and diagnostic biomarkers. Visualization of the correlation results was conducted employing the R package ggplot2.

### 2.7 Gene set enrichment analysis

Gene set enrichment analysis (GSEA) was conducted utilizing both the R package clusterProfiler and R package enrichplot to investigate signaling pathways associated with unstable plaques. The gene set “c2.cp.kegg.v7.4.symbols.gmt” from the Molecular Signatures Database (MsigDB) served as the background gene set. Enrichment results with a *p*-value < 0.05 and *q*-value < 0.05 were deemed statistically significant.

### 2.8 Immunohistochemical staining

The expression of target proteins in coronary atherosclerotic plaque tissues was assessed via immunohistochemical staining. Sections measuring 5 μm were sliced from paraffin-embedded coronary artery tissues. Deparaffinization of xylene was followed by hydration with an ethanol concentration gradient. Microwave heating was employed to facilitate antigen retrieval. Endogenous peroxidase activity was inhibited using a 3% hydrogen peroxide solution. Samples were blocked with a 5% goat serum solution. Sections were then incubated overnight at 4°C with anti-HSPA2 anti-body (1:200, Proteintech, USA) and anti-GEM antibody (1:200, GeneTex, USA), applied dropwise. The next day, sections were washed with phosphate-buffered saline (PBS) and subsequently incubated with horseradish peroxidase (HRP)-labeled goat anti-rabbit secondary antibody, applied dropwise, for 30 min at 37°C. Color development was achieved using diaminobenzidine (DAB), followed by counterstaining with hematoxylin. Following dehydration with xylene and ethanol gradient, the slides were sealed with neutral gum. Positive expression of the target protein was visualized as brown staining under the microscope. Three fields of view were chosen, and photographs were captured at a magnification of 200×. ImageJ software was utilized to quantify the average optical density (AOD) of the positive signals (positive expression optical density value divided by the total measured area). The results were averaged from three measurements conducted in triplicate.

### 2.9 Statistical analysis

Data processing and statistical analyses were primarily conducted using R (version 4.3.1) and GraphPad Prism (version 9.0.0). Expression differences among subgroups were evaluated using the Wilcoxon test. Correlation analyses were conducted using the Spearman correlation test. Immunohistochemical measurements were presented as mean ± standard deviation (mean ± SD) and assessed for normal distribution and variance homogeneity. Unpaired t-tests were conducted for data with normal distribution and homogenous variance.

## 3 Results

### 3.1 Screening of DEGs between unstable and stable plaques

**Figure 1:**
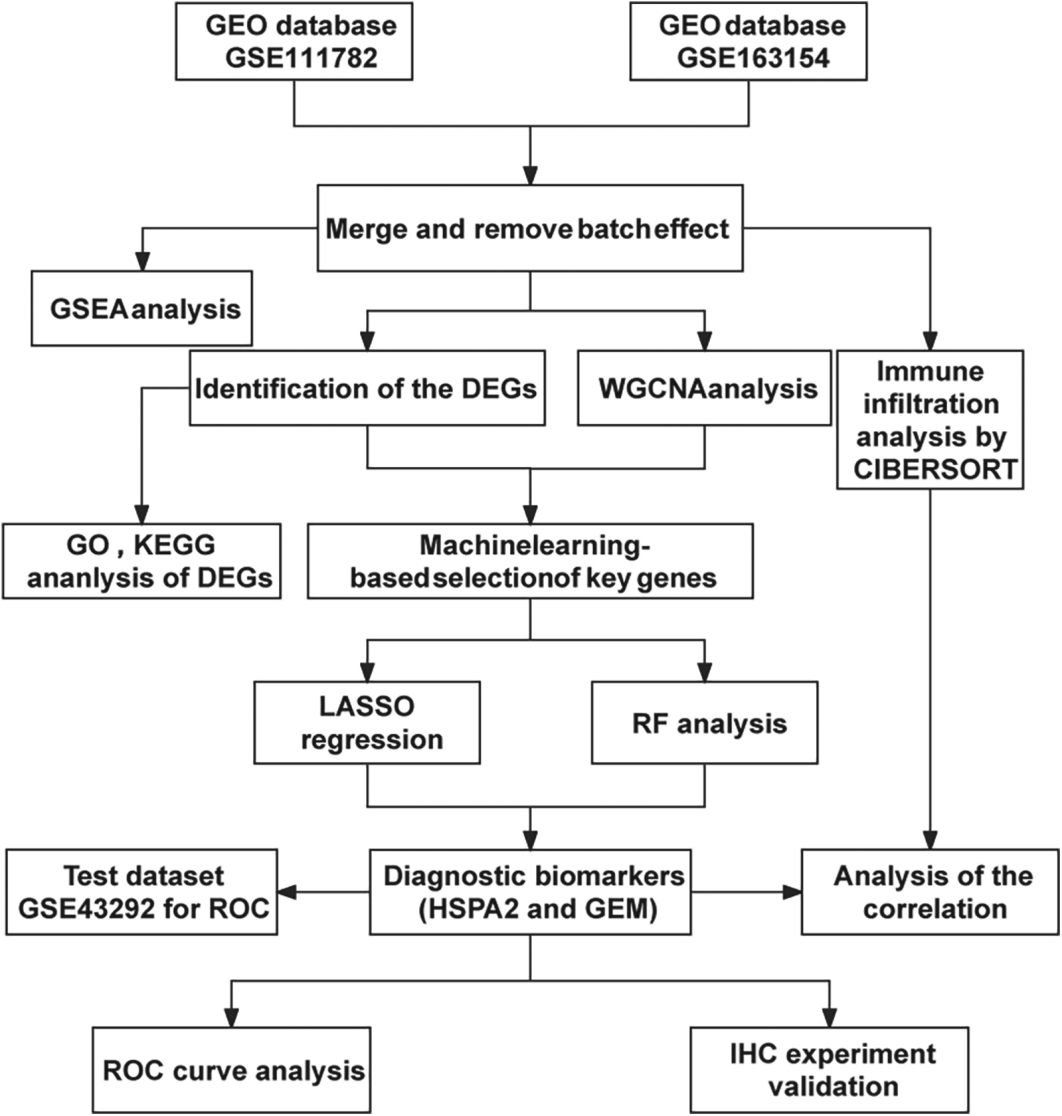
Flow chart.

**Figure 2:**
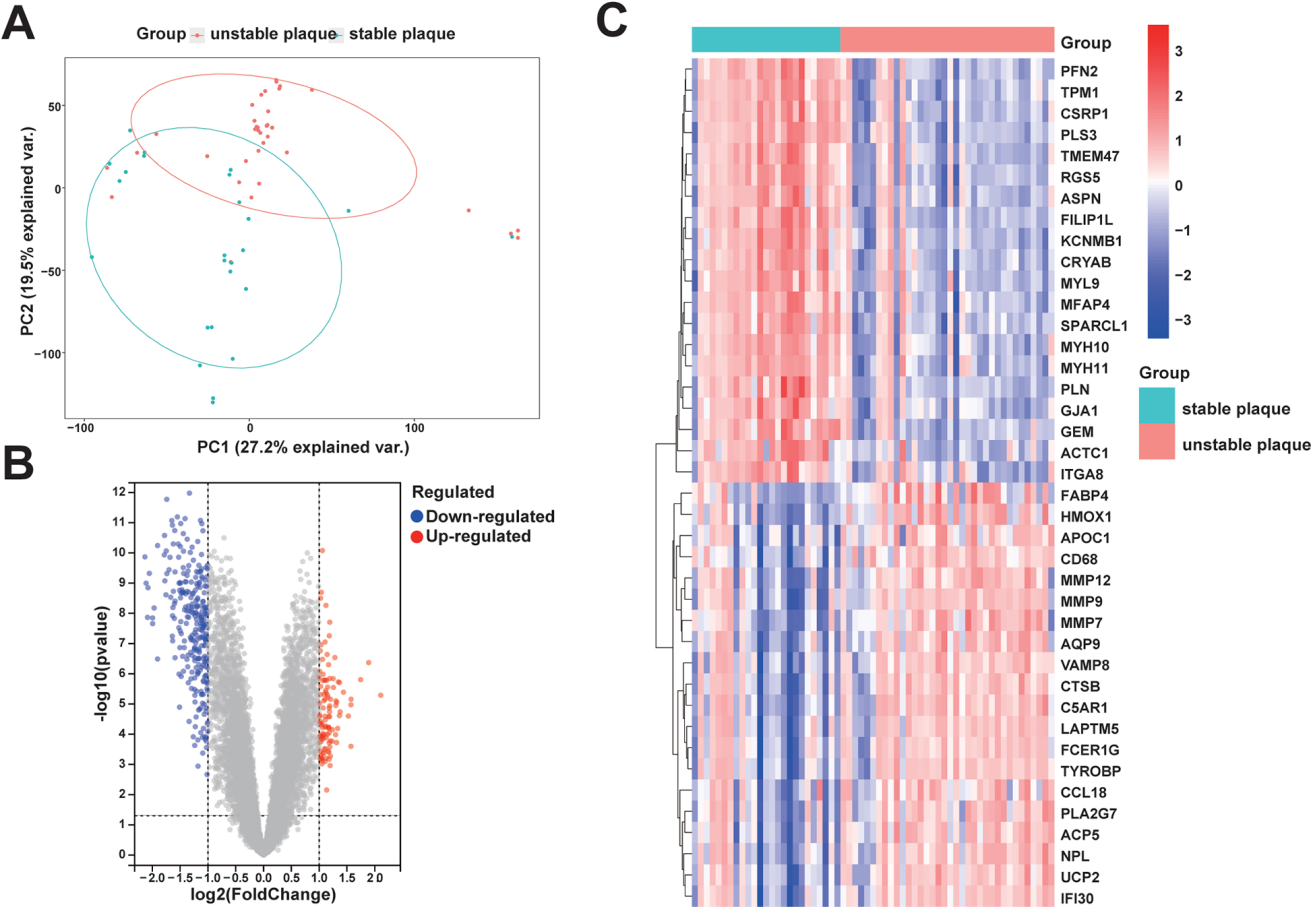
PCA and DEGs identification between unstable and stable plaque subgroups: **(A)** PCA plot of the merged dataset; **(B)** volcano plot of DEGs between unstable and stable plaque subgroups. Blue dots represent DEGs with down-regulated expression and red dots represent DEGs with up-regulated expression; **(C)** heatmap of relative expression levels of some DEGs.

The analysis workflow in this study is depicted in Figure 1. We examined the disparities in gene expression between the unstable plaque group and the stable plaque group. Principal component analysis (PCA) of the normalized gene expression matrix (Figure 2A) revealed significant disparities between the unstable and stable plaque groups. This led to the identification of 384 significantly DEGs associated with unstable plaques under the screening criteria of *p*-value < 0.05. Among these were 273 genes exhibiting down-regulated expression and 111 genes demonstrating up-regulated expression (Figures 2B,C).

### 3.2 Functional enrichment analysis of DEGs

**Figure 3:**
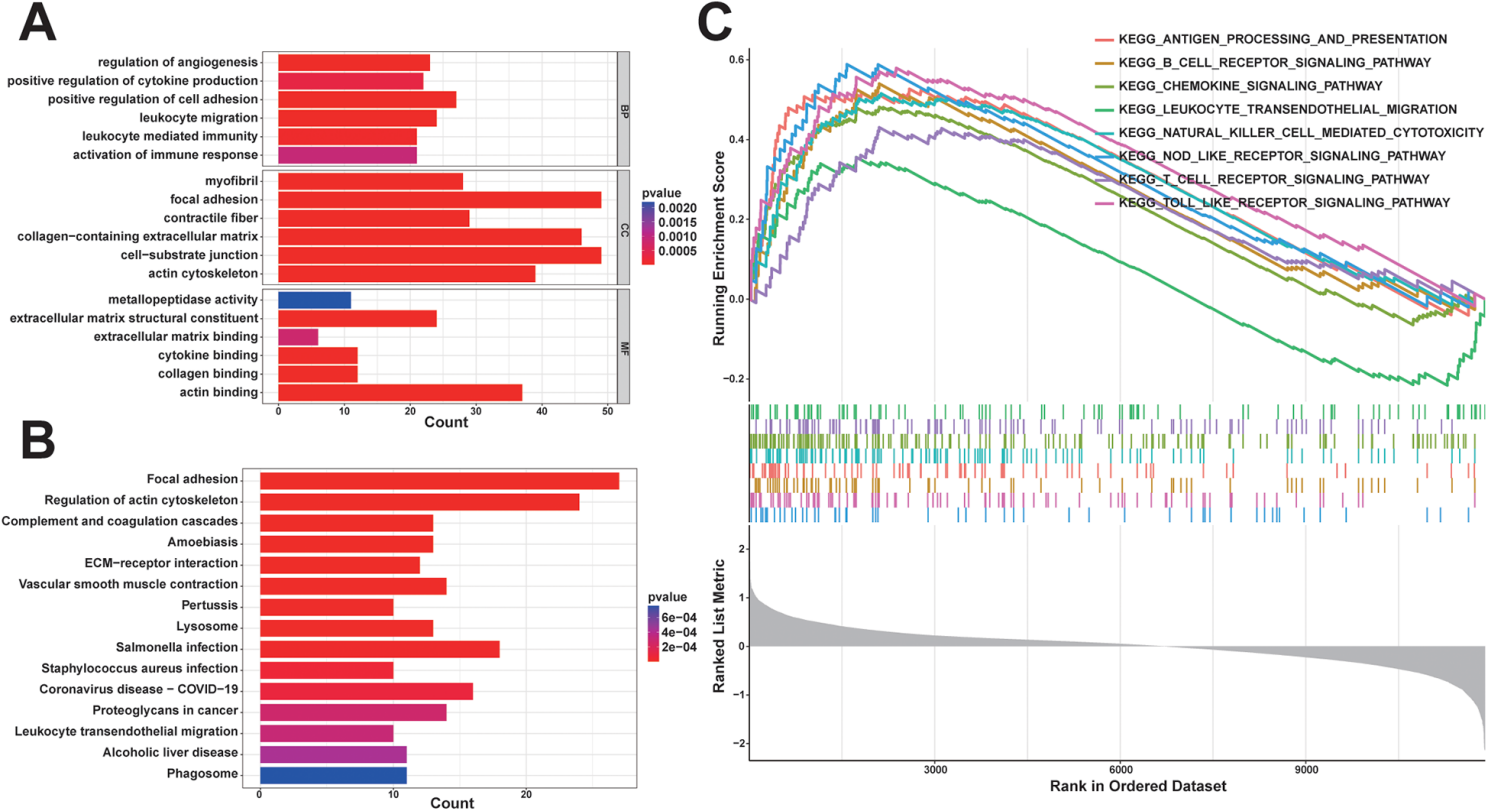
Functional enrichment analysis: **(A)** some representative enrichment results in GO enrichment analysis of DEGs; **(B)** KEGG enrichment analysis results of DEGs; **(C)** some representative significant enrichment pathways of GSEA-KEGG.

In order to comprehensively understand the biological functions and distribution of DEGs among different subgroups, we conducted GO and KEGG enrichment analyses of the DEGs. The GO enrichment analyses revealed that the DEGs were predominantly enriched in processes such as positive regulation of cell adhesion, leukocyte migration, regulation of angiogenesis, positive regulation of cytokine production, leukocyte-mediated immunity, activation of the immune response, collagen-containing extracellular matrix, and extracellular matrix structural constituent (Figure 3A). Furthermore, the KEGG enrichment analysis indicated that the DEGs were enriched in pathways related to leukocyte transendothelial migration and hematopoietic cell lineage (Figure 3B). These enrichment results suggest that the diminished stability of atherosclerotic plaques may be closely associated with immune cell migration and activation, extracellular matrix production, lipid metabolism, and neovascularization within plaques.

### 3.3 The results of GSEA

In order to explore potential pathways associated with unstable plaques during the progression of coronary atherosclerotic plaques, we utilized the background gene set provided by the MsigDB database for GSEA. Pathways with |normalized enrichment score (NES)∣>1, a *p*-value < 0.05, and a *q*-value < 0.05 were generally considered as significantly enriched. The findings indicated that KEGG signaling pathways significantly enriched in unstable plaques compared to stable plaques primarily consisted of inflammatory and immune-related pathways, including the toll-like receptor signaling pathway, B cell receptor signaling pathway, antigen processing and presentation, cytokine-cytokine receptor interaction, chemokine signaling pathway, and T cell receptor signaling pathway (Figure 3C). These results suggest that immune and inflammatory signaling pathways are associated with unstable atherosclerotic plaques.

### 3.4 Construction of weighted gene co-expression network and identification of key modules

**Figure 4:**
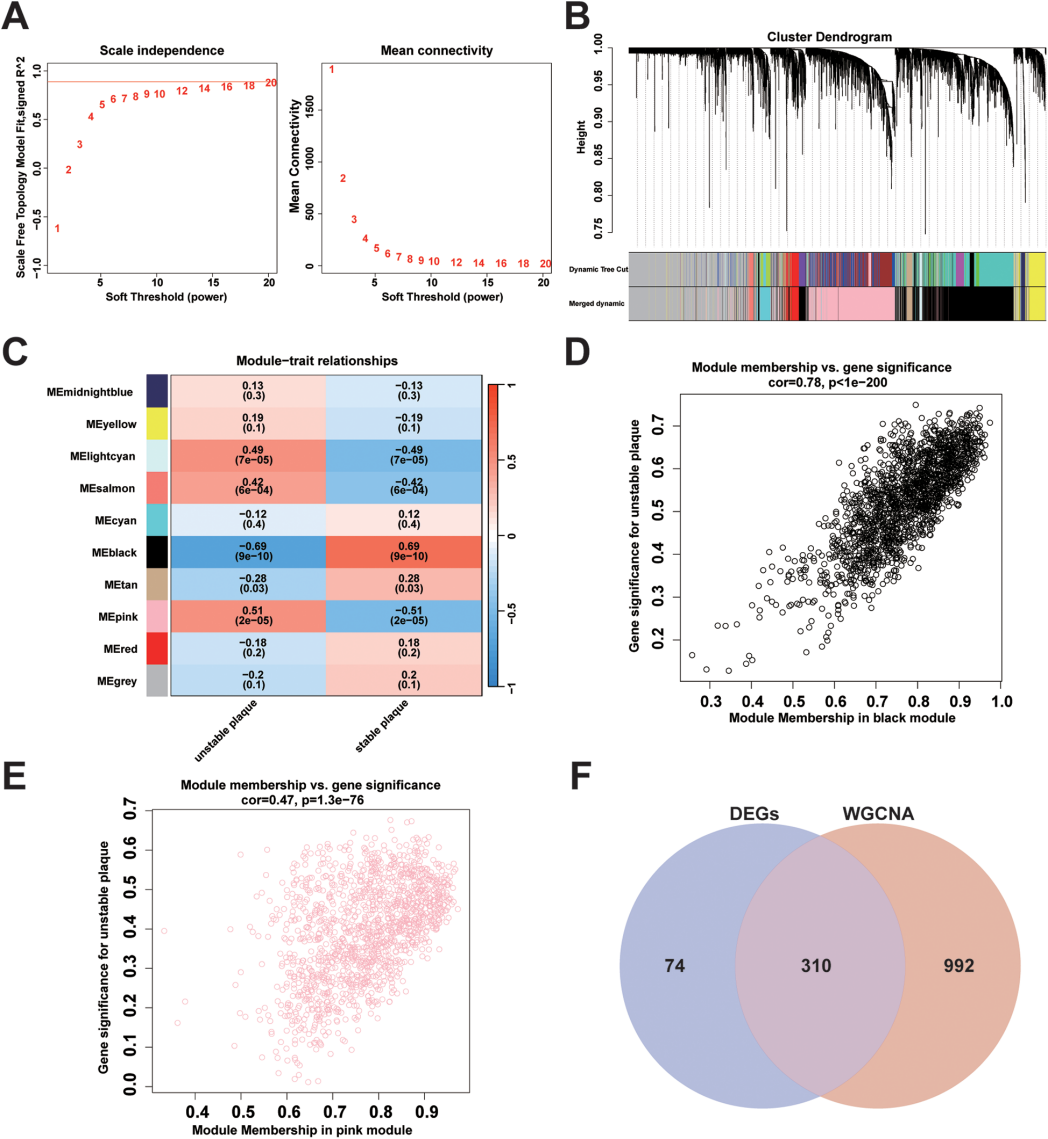
Weighted gene co-expression network construction and identification of key modules: **(A)** analysis of changes in scale-free topological fit index (left) and average connectivity (right) for different soft thresholds (β); **(B)** gene hierarchy clustering dendrogram. Different colours represent different co-expression modules; **(C)** heatmap of module eigengene correlation with traits. The black module and the pink module were significantly correlated with traits; **(D)** scatterplot of correlation between MM and GS of genes in the black module; **(E)** scatterplot of correlation between MM and GS for genes in the pink module; **(F)** Venn plots showing the intersection of key module genes with DEGs obtained from WGCNA analysis.

Genes in the top 50% of MAD from the merged dataset were selected to construct a weighted gene co-expression network. All samples were clustered and observed without significant outlier samples. The optimal soft threshold *β* = 20 (scale-free *R*^2^ = 0.85, mean connectivity = 2.41) was chosen to construct the scale-free topological network (Figure 4A). After clustering genes with similar expression patterns, modules were identified using the dynamic tree-cutting algorithm, and related modules were merged to obtain a total of 10 modules (Figure 4B). A heat map illustrating module-trait correlations revealed significant associations between the black module (cor = −0.69, *p* = 9e-10) and the pink module (cor = 0.51, *p* = 2e-05) with unstable plaques (Figure 4C). Furthermore, scatter plots depicted a strong correlation between genes MM and GS in the black module (cor = 0.78, *p* < 1e-200) (Figure 4D) and an equally strong correlation between genes MM and GS in the pink module (cor = 0.47, *p* = 1.3e-76) (Figure 4E). Based on the screening criteria of MM >0.8 and GS >0.2, a total of 739 key module genes were identified in the black module, and 563 in the pink module.

### 3.5 Screening for diagnostic markers using machine learning algorithms

**Figure 5:**
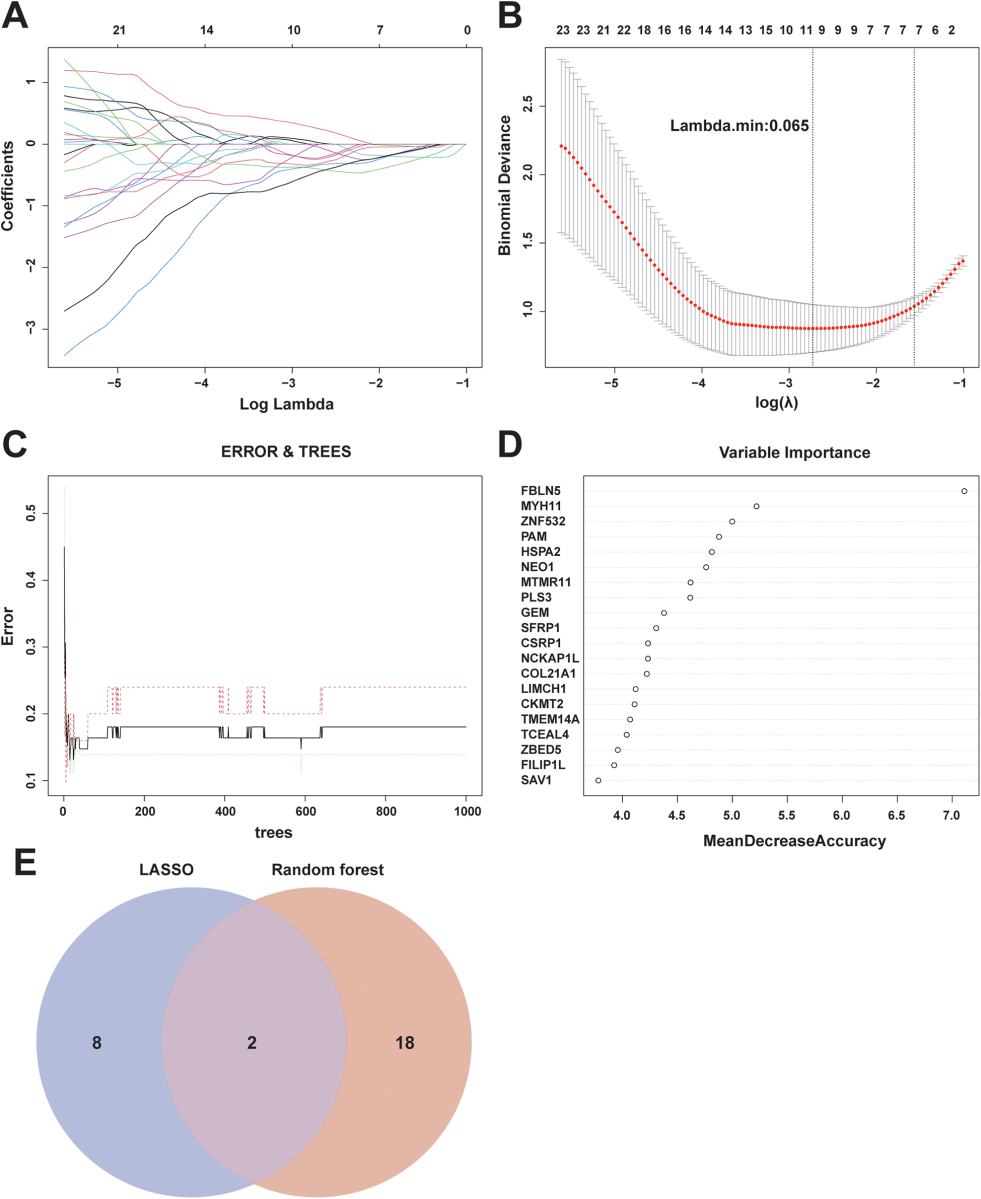
Screening of diagnostic markers using machine learning algorithms: **(A)** regression coefficient curves of genes in LASSO regression analysis; **(B)** selection of optimal tuning parameter (λ) based on ten-fold cross-validation of binomial deviance; **(C)** analysis of model error rate of RF algorithm in relation to the number of decision trees; **(D)** RF model average accuracy reduction ranking top 20 feature genes; **(E)** Venn diagram showing the intersection of the screening results of two machine learning algorithms.

A total of 310 overlapping genes were identified through the use of a Venn diagram to determine the intersection between DEGs and key module genes (Figure 4F). Subsequently, two machine learning algorithms, LASSO regression analysis and RF, were employed to identify diagnostic biomarkers associated with unstable plaques. In LASSO regression analysis, an L1 penalty term λ was introduced, resulting in model regression coefficients gradually converging to 0 as the value of λ increased (Figure 5A). Ten feature genes were selected based on ten-fold cross-validation, determined by selecting the lambda value corresponding to the minimum average cross-validation error (Figure 5B). The RF algorithm first determines the number of decision trees for constructing the model based on the error rate (Figure 5C). Then, the top 20 genes are selected in order of importance assessed by the MDA index as the feature genes (Figure 5D). The intersection of the feature genes identified by the two aforementioned machine learning algorithms led to the identification of HSPA2 and GEM as key genes (Figure 5E).

### 3.6 Assessment of diagnostic efficacy of diagnostic biomarkers

**Figure 6:**
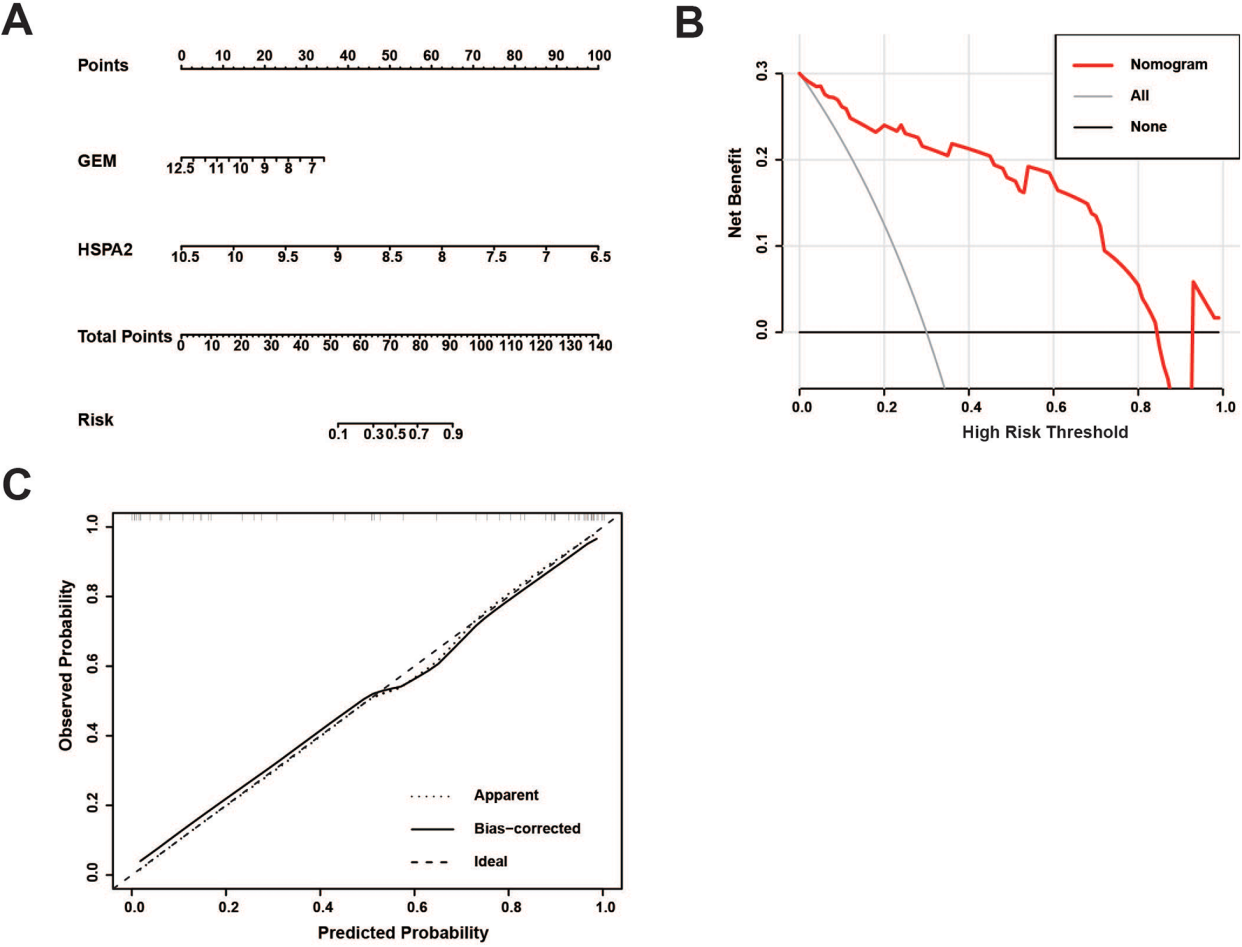
Development of a nomogram model for predicting the probability of unstable plaque formation: **(A)** prediction of unstable plaque occurrence using the nomogram; **(B)** clinical decision curve analysis for evaluating the potential benefits of predictive modeling in clinical practice; **(C)** calibration curve is utilized to evaluate the alignment between the predicted probabilities of a model and the actual observations.

**Figure 7:**
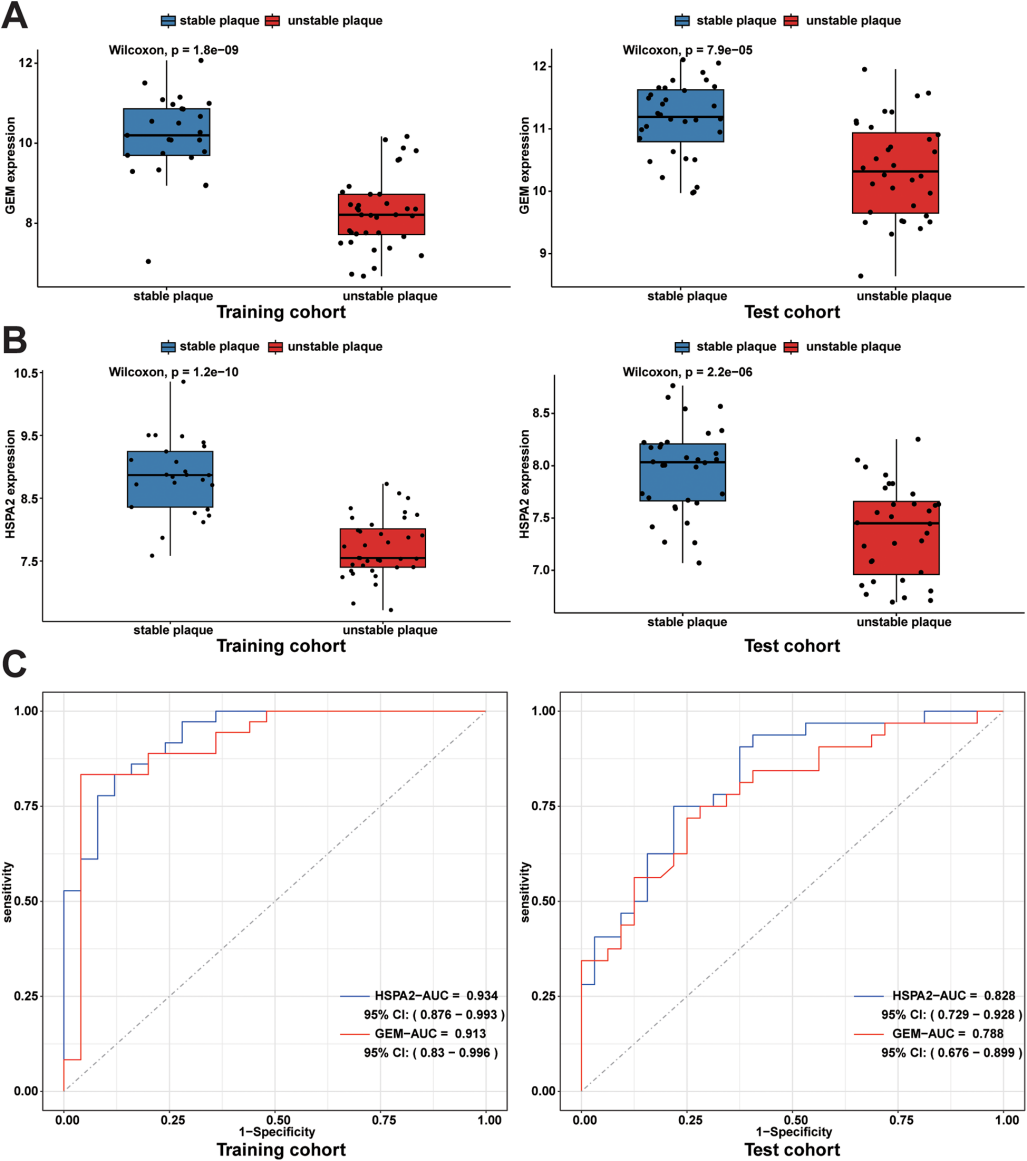
Expression levels of diagnostic biomarkers and assessment of diagnostic efficacy in the training and test cohorts: **(A)** box line plots of GEM expression in unstable plaque subgroups and stable plaque subgroups in the training cohort and test cohort; **(B)** box plots of HSPA2 expression in unstable and stable plaque subgroups in the training and test cohorts; **(C)** ROC curves for diagnostic efficacy assessment of HSPA2 and GEM in the training cohort and test cohort.

An independent dataset, GSE43292, comprising 32 stable plaque samples and 32 unstable plaque samples, was utilized as the test set. The Wilcoxon rank-sum test results indicated significantly higher expression levels of HSPA2 and GEM in the stable plaque group compared to the unstable plaque group in the training cohort (*p* < 0.001). We constructed nomogram for the diagnosis of unstable plaques, which demonstrates the effect of each predictor variable on the probability of unstable plaque formation. Based on the results of the DCA, the nomogram model demonstrated a substantial net benefit across most threshold probabilities, indicating the model's substantial value in clinical decision-making (Figures 6A,B). Additionally, the calibration curve shows that the model has good predictive accuracy (Figure 6C). The differences in expression levels between the HSPA2 and GEM groups were corroborated in the test cohort, aligning with the findings of the training cohort (Figures 7A,B). Subsequently, we evaluated the diagnostic efficacy of HSPA2 and GEM for unstable plaques by constructing ROC curves and measuring the AUC. Within the training cohort, HSPA2 (AUC = 0.934) and GEM (AUC = 0.913) exhibited promising diagnostic efficacy for unstable plaques. Similarly, in the test cohort, HSPA2 (AUC = 0.828) and GEM (AUC = 0.788) demonstrated favorable diagnostic efficacy for unstable plaques (Figure 7C).

**Figure 8:**
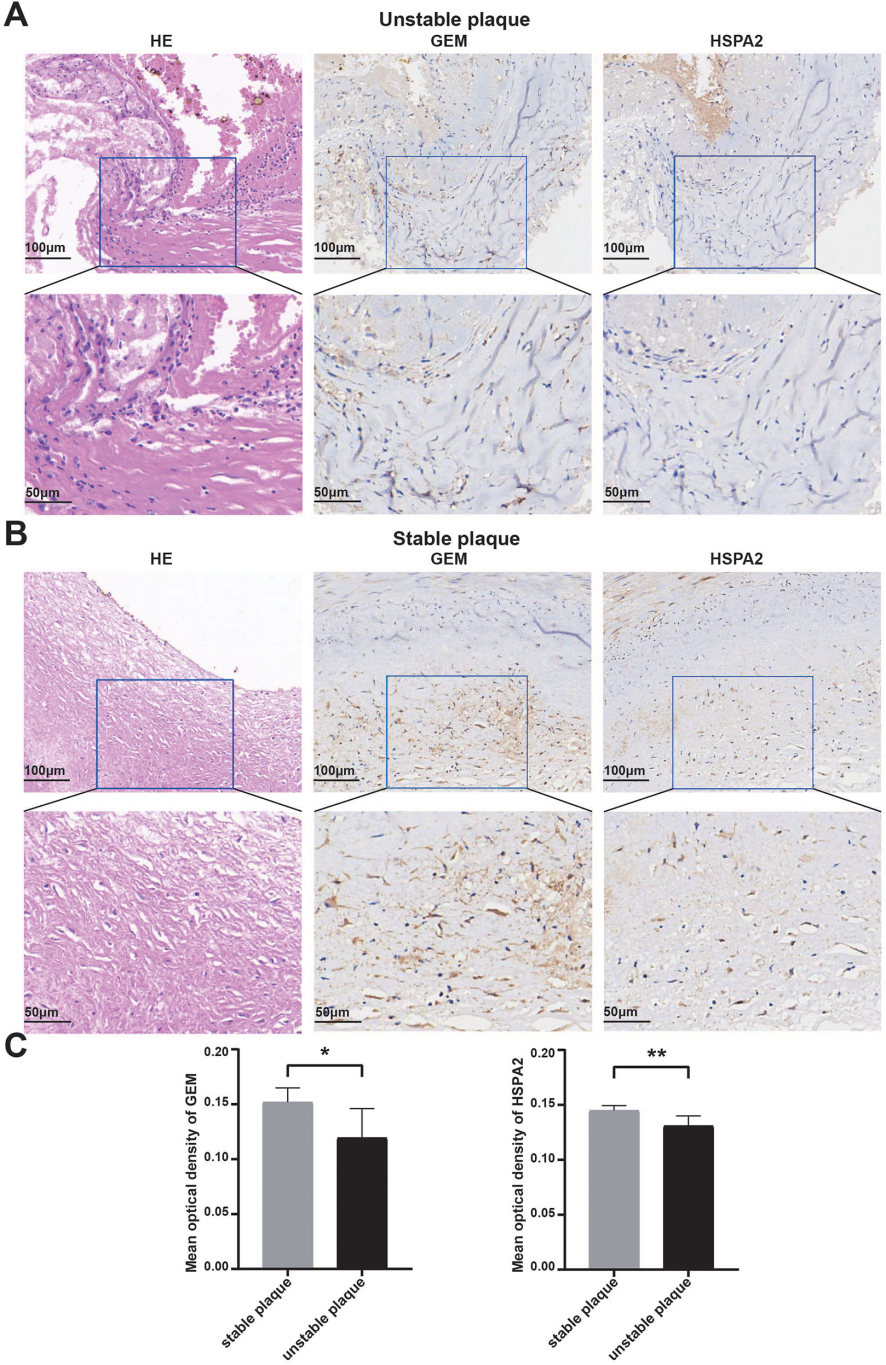
Validation of diagnostic biomarkers by human tissue samples: **(A,B)** representative images of hematoxylin-eosin staining of unstable coronary atherosclerotic plaques and stable coronary atherosclerotic plaques and IHC staining of GEM and HSPA2 proteins; **(C)** results of quantitative analysis of GEM and HSPA2 in different subgroups. Data were expressed using mean ± SD. * represents <0.05, ** represents <0.01.

To bolster the validation of the diagnostic efficacy of the identified biomarkers for unstable plaques, we assessed the stability of acquired human coronary atherosclerotic plaques utilizing the AHA Atherosclerotic Plaque Stability Scoring System (Supplementary Table 1). Immunohistochemical staining was conducted, followed by the measurement of mean optical density values of positive protein expression using ImageJ software. The results revealed a concordance with the bioinformatics analysis, indicating elevated expression levels of HSPA2 and GEM in stable plaques and their downregulation in unstable plaques (Figures 8A,B). Additionally, the disparity between the two groups was statistically significant (Figure 8C).

### 3.7 Analysis of immune cell infiltration and its correlation with diagnostic biomarkers

**Figure 9:**
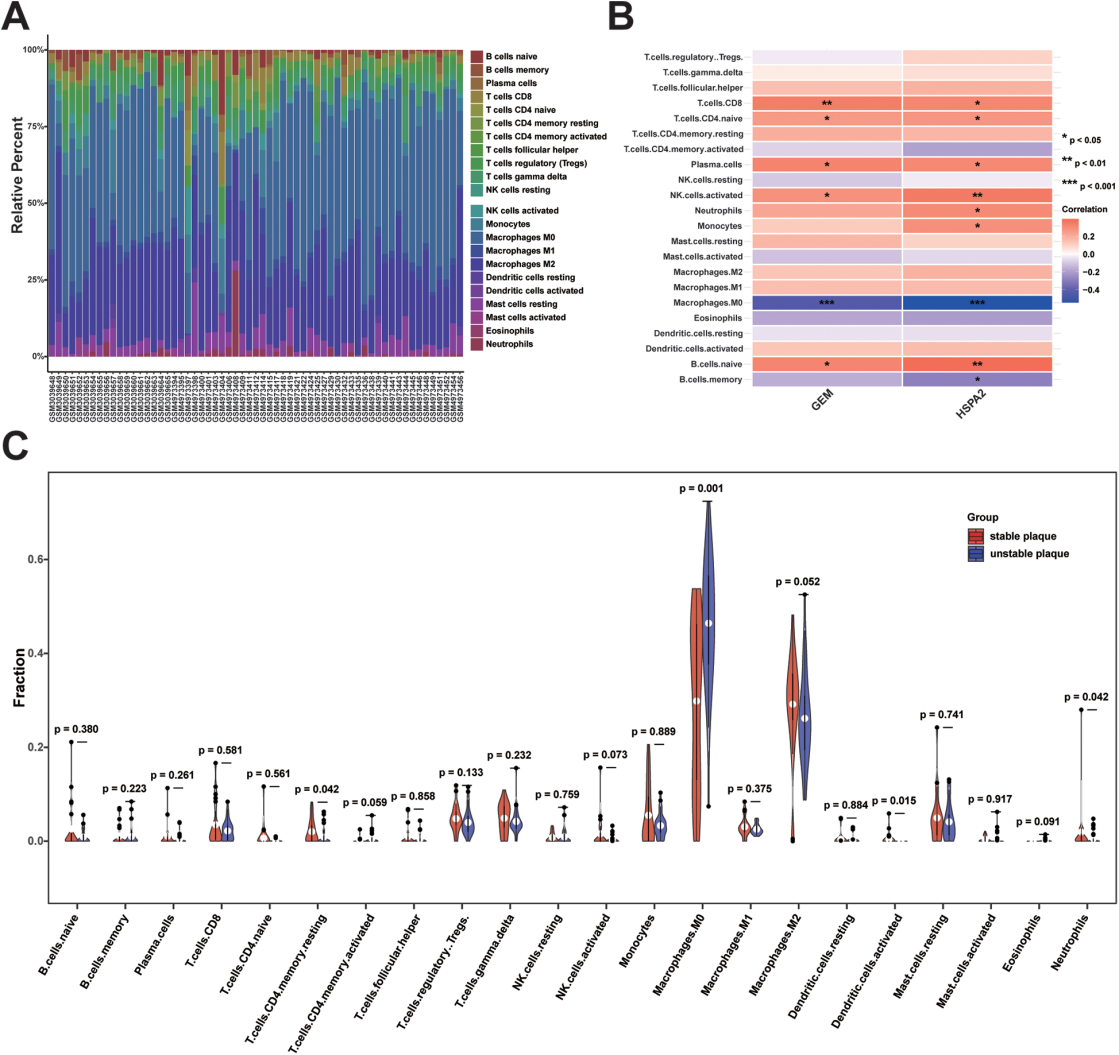
Analysis of immune cell infiltration between unstable and stable plaques: **(A)** bar graph of the proportion of 22 immune cell infiltrates in unstable vs. stable plaques; **(B)** heatmap of correlation analysis between diagnostic biomarkers and immune cell infiltration; **(C)** violin plot of difference analysis of immune cell infiltration between unstable and stable plaques.

To investigate disparities in immune cell infiltration between stable and unstable plaque groups, we conducted immune cell infiltration analysis utilizing the CIBERSORT algorithm. The results of the CIBERSORT analysis revealed 22 immune cell sub-populations in 61 samples. Among these, Naïve B cells, gamma delta T cells (γδ T cells), Tregs, M0 macrophages, M2 macrophages, and resting mast cells predominated in atherosclerotic plaques (Figure 9A). The proportion of M0 macrophage infiltration was significantly higher in unstable plaques compared to stable plaques (*p* < 0.05). Conversely, the proportions of resting CD4 + memory T cells, activated dendritic cells, and neutrophils were relatively elevated in stable plaques (*p* < 0.05) (Figure 9C). Additionally, correlation analysis between diagnostic biomarkers and immune cells revealed significant positive correlations of CD8+ T cells, naïve CD4+ T cells, plasma cells, activated natural killer cells, and naïve B cells with both GEM and HSPA2, whereas M0 macrophages exhibited significant negative correlations with both GEM and HSPA2 (Figure 9B). These findings suggest a potential role of immune cell infiltration in the formation of unstable plaques.

## 4 Discussion

Presently, due to the widespread adoption of the concept of healthy living and the availability of numerous clinical treatments, the risk of cardiac mortality and myocardial infarction among patients with coronary artery disease has decreased. However, a subset of patients with coronary atherosclerosis experiences a decline in plaque stability, leading to the progression of ACS. For instance, among patients with diabetes and those presenting initially with ACS, the incidence of cardiac death and myocardial infarction remains elevated despite optimal pharmacological treatment and successful revascularization of the stenotic vessel (24). Coronary atherosclerosis constitutes a group of chronic inflammatory diseases, where alterations in plaque necrotic core size and fibrous cap thickness, influenced by immune cell infiltration and cytokine expression within plaques, impact plaque stability (9, 25–27). Hence, to enhance the prognosis of cardiovascular disease, it is imperative to identify specific diagnostic biomarkers of unstable coronary atherosclerotic plaques and explore the pattern of immune cell infiltration associated with unstable plaques.

Utilizing data from public databases, the study identified 384 significant DEGs between unstable and stable plaque subgroups, comprising 273 down-regulated genes and 111 up-regulated genes. Subsequent GO enrichment analysis unveiled that the DEGs were primarily associated with processes such as leukocyte migration, regulation of angiogenesis, leukocyte-mediated immunity, and activation of the immune response. These processes suggest the dynamic involvement of immune and inflammatory responses in the development and destabilization of atherosclerotic plaques. The migration of leukocytes, for instance, is a hallmark of inflammatory responses, which contribute to plaque instability by enhancing the recruitment of immune cells to the plaque site. KEGG enrichment analysis unveiled that the DEGs were enriched in inflammatory and immune-related pathways, including pathways related to Staphylococcus aureus infection, leukocyte transendothelial migration, and hematopoietic cell lineage. These pathways highlight the central role of immune dysregulation in plaque instability. In particular, leukocyte transendothelial migration is a critical step in the accumulation of immune cells within the plaque, where they can exacerbate local inflammation and contribute to the formation of a necrotic core—a feature associated with vulnerable plaques. Furthermore, the involvement of hematopoietic cell lineage pathways suggests that bone marrow-derived cells may contribute to plaque destabilization through their differentiation into pro-inflammatory subsets that further promote plaque progression. Building upon these findings, GSEA was conducted, revealing significant enrichment of pathways such as the B cell receptor signaling pathway, antigen processing and presentation, cytokine-cytokine receptor interaction, chemokine signaling pathway, and T cell receptor signaling pathway in the unstable plaque subgroup. These pathways are particularly relevant for understanding the immune interactions that drive plaque instability. For instance, the B cell receptor signaling pathway and antigen processing and presentation pathways highlight the role of adaptive immunity, including the activation of B cells and T cells, in the response to the atherosclerotic plaque. These immune cells, particularly T lymphocytes, are known to infiltrate plaques and can promote either pro-inflammatory or anti-inflammatory responses, depending on the plaque's stage and the local environment. The chemokine signaling pathway and T cell receptor signaling pathway, on the other hand, emphasize the importance of chemokines in directing immune cell migration and the role of T cells in sustaining chronic inflammation within the plaque. Inflammatory cytokines released by T cells and other immune cells can further disrupt plaque integrity, leading to a thin fibrous cap, a hallmark of unstable plaques. Consistent with prior research (14, 25–27), these pathways play pivotal roles in the progression of coronary atherosclerotic plaques. Intimal retention of LDL at sites of coronary endothelial dysfunction facilitates the adherent infiltration of inflammatory cells into the plaque, while atherosclerosis, characterized by a chronic inflammatory response, attracts chemotactic infiltration of innate and adaptive immune cells, primarily comprised of T lymphocytes (28). Certain cells recognize ApoB, the core protein of LDL particles, and differentiate to generate distinct cell subtypes that elicit either pro- or anti-atherosclerotic effects (29–32). These biological processes elucidate the potential mechanisms influencing plaque stability.

Leveraging the aforementioned DEGs, WGCNA, and two machine learning algorithms were integrated to screen and identify potential diagnostic biomarkers for unstable coronary atherosclerotic plaques. Being a method in systems biology analysis, WGCNA was employed to detect gene modules exhibiting highly correlated expression patterns. Gene networks were constructed using the optimal soft threshold β to conform to the scale-free network distribution. The hierarchical clustering method and dynamic tree cutting algorithm were utilized to derive gene modules, wherein genes exhibit strong functional correlations. Through correlating modules with disease phenotypes, two modules, namely the black and pink modules, were identified as significantly associated with unstable plaques. The black and pink modules exhibited negative and positive correlations with unstable plaques, respectively. Gene expression within the black module potentially inhibits the progression of unstable plaques, while gene expression within the pink module may facilitate unstable plaque formation. The RF algorithm, an integrative learning technique in machine learning, employs bagging with random sampling to construct decision trees, which are then integrated into random forest for final decision-making through voting. This algorithm offers a feature selection mechanism by assessing the impact of feature genes on the accuracy of the RF model (33, 34). LASSO regression, a linear regression technique, operates on the principle of introducing an L1 penalty term derived from the ordinary least squares method. This method accomplishes feature selection by nullifying coefficients of redundant features to zero (35). Leveraging the aforementioned machine learning algorithms, we conducted further screening to identify diagnostic biomarkers for unstable plaques, resulting in the selection of HSPA2 and GEM.

The validation of these genes was further conducted using the test dataset. Significant differences in the expression levels of HSPA2 and GEM were observed between the unstable and stable plaque groups. ROC curves demonstrated the favorable diagnostic efficacy of both HSPA2 and GEM. HSPA2 belongs to the Heat Shock Protein 70 kDa (HSP70) family (36). Experimental evidence suggests that HSP70 plays a role in the pathogenesis of atherosclerosis and possesses a protective effect in cardiovascular disease. Elevated serum levels of HSP70 are associated with a reduced risk of coronary artery disease (37–42). González-Ramos et al. reported that HSP70 is associated with a reduced risk of coronary artery disease through the upregulation of TGF-β1, which promotes extracellular matrix expression by smooth muscle cells in blood vessels (43). This finding aligns with our conclusion that HSPA2 potentially contributes to the stabilization of coronary atherosclerotic plaques. Despite limited exploration in cardiovascular disease thus far, GEM exhibits potential as a promising novel therapeutic target pending further validation.

In this study, we employed the CIBERSORT algorithm to analyze the gene expression matrix (44), aiming to estimate the abundance of various immune cell infiltrates in coronary atherosclerotic plaques and to deepen our understanding of their impact on plaque progression. Variations in immune cell infiltration levels may correlate with plaque instability. Our findings revealed that γδ T cells, Tregs, M0 macrophages, and M2 macrophages constituted the predominant immune cell populations within atherosclerotic plaques, aligning with prior research highlighting T cells and macrophages as the predominant leukocyte types in such plaques (28, 45, 46). Elevated levels of M0 macrophage infiltration were observed in unstable plaques compared to stable plaques. Additionally, dysfunctional coronary artery endothelial cells expressed vascular cell adhesion molecules and cytokines to facilitate monocyte recruitment to the intima (47–49). Monocytes undergo differentiation into macrophages under the stimulation of macrophage colony-stimulating factor (M-CSF) synthesized within the local intima (50, 51). Activated macrophages secrete pro-inflammatory cytokines, including IL-1β and TNF-α, which enhance inflammatory cell adhesion and aggregation, amplifying the local inflammatory response at the lesion site and inducing the expression of MMPs (15, 52). This process diminishes the strength of the plaque fibrous cap, thereby impacting plaque stability. Correlation analysis revealed associations between GEM and HSPA2 expression and the infiltration of various immune cell types, such as CD8+ T cells, naïve CD4+ T cells, plasma cells, activated natural killer cells, naïve B cells, and M0 macrophages. Thus, GEM and HSPA2 may synergistically influence plaque stability in concert with immune cells. It is important to note that transcriptome sequencing may not fully capture the immune cell phenotype. Subsequent investigations could integrate cell surface markers and single-cell transcriptome analysis to achieve a more comprehensive understanding of immune cell infiltration in atherosclerotic plaques.

This study exhibits several limitations. Initially, microarray datasets from multiple independent studies were amalgamated in this investigation. Despite normalization efforts to mitigate batch effects, heterogeneity persists as an inevitable challenge that could potentially influence the analysis outcomes. Secondly, limitations in drawing conclusions may arise from the small number of coronary artery tissue samples utilized for experimental validation. Furthermore, in contrast to quantitative gene expression assays, such as protein imprinting, immunohistochemistry, while providing valuable insights into protein localization and semi-quantitative expression, has its own inherent limitations. This technique is prone to observer variability, and its semi-quantitative nature may introduce discrepancies in measurement results. Additionally, in clinical practice, obtaining coronary atherosclerotic plaque tissue samples is challenging, often requiring invasive procedures such as coronary endarterectomy or intravascular ultrasound-guided plaque sampling. These procedures are not only risky but also traumatic for patients, limiting their widespread use as routine tests in clinical practice. This restricts the direct application and translation of relevant research findings into clinical diagnosis and treatment. Future research could explore the potential of circulating biomarkers in peripheral blood as indicators of the biological status of plaques, addressing the limitations of the current study and facilitating the practical application of the findings in clinical settings.

## 5 Conclusions

Through the integration of WGCNA with machine learning algorithms, we identified HSPA2 and GEM as potential diagnostic biomarkers for the early detection of unstable coronary atherosclerotic plaques. Moreover, the findings suggest that immune cell infiltration might influence coronary atherosclerotic plaque stability. Furthermore, HSPA2 and GEM showed significant associations with a variety of immune cells. These findings offer new insights into both the pattern of immune infiltration in unstable coronary atherosclerotic plaques and their underlying immunoregulatory mechanisms.

## Data Availability

Publicly available datasets were analyzed in this study. This data can be in the Gene Expression Omnibus database at https://www.ncbi.nlm.nih.gov/geo/, accession numbers: GSE111782, GSE163154 and GSE43292.
